# Retraction Note: Comparison of safety and effectiveness of antiretroviral therapy regimens among pregnant women living with HIV at preconception or during pregnancy: a systematic review and network meta-analysis of randomized trials

**DOI:** 10.1186/s12879-026-13152-6

**Published:** 2026-03-27

**Authors:** Fatemeh Mehrabi, Mohammad Karamouzian, Behnam Farhoudi, Shahryar Moradi Falah Langeroodi, Soheil Mehmandoost, Samaneh Abbaszadeh, Shahrzad Motaghi, Ali Mirzazadeh, Behnam Sadeghirad, Hamid Sharifi

**Affiliations:** 1 HIV/STI Surveillance Research Center, WHO Collaborating Center for HIV, Surveillance Institute for Futures Studies in Health, Kerman University of Medical Sciences, Kerman, Iran; 2 Centre on Drug Policy Evaluation, MAP Centre for Urban Health Solutions, St. Michael’s Hospital, Toronto, ON Canada; 3 Dalla Lana School of Public Health, University of Toronto, Toronto, ON Canada; 4 Social Determinants of Health Research Center, Amir-al-momenin Hospital, Tehran Medical Sciences, Islamic Azad University, Tehran, Iran; 5 Pharmaceutics Research Center, Institute of Neuropharmacology, Kerman University of Medical Sciences, Kerman, Iran; 6 Department of Health Research Methods, Evidence, and Impact (HEI), McMaster University, Hamilton, ON Canada; 7 Department of Epidemiology and Biostatistics, University of California San Francisco, San Francisco, CA USA; 8 Department of Anesthesia, McMaster University, Hamilton, ON Canada; 9 Michael G. DeGroote Institute for Pain Research and Care, McMaster University, Hamid Sharifi, Hamilton, ON Canada; 10 Institute for Global Health Sciences, University of California, San Francisco, San Francisco, CA USA

**Retraction Note: BMC Infectious Diseases (2024) 24:417**

10.1186/s12879-024-09303-2

The Editors have retracted this article. After publication, concerns were raised regarding the article selection for the study of antiretroviral treatment regimens presented in this review. Specifically, the EFV/TDF/FTC and EFV/ZDV/3TC regimens are wrongly considered to be equivalent, which resulted in the incorrect inclusion of treatment studies. The Editors have determined that these errors invalidate the conclusions of this review.

Hamid Sharifi, Behnam Farhoudi, Mohammad Karamouzian, Soheil Mehmandoost, Fatemeh Mehrabi, Ali Mirzazadeh, Shahryar Moradi, Shahrzad Motaghi, and Behnam Sadeghirad disagree with this retraction. The Publisher was unable to contact Samaneh Abbaszadeh.

